# *N*-Cyclo­hexyl-2-fluoro­benzamide

**DOI:** 10.1107/S1600536808034090

**Published:** 2008-10-25

**Authors:** Aamer Saeed, Rasheed Ahmad Khera, Naeem Abbas, Jim Simpson, Roderick G. Stanley

**Affiliations:** a Department of Chemistry, Quaid-i-Azam University, Islamabad 45320, Pakistan; b Department of Chemistry, University of Otago, PO Box 56, Dunedin, New Zealand

## Abstract

In the title compound, C_13_H_16_FNO, the fluoro­benzene ring plane and the plane through the amide unit are inclined at a dihedral angle of 29.92 (7)°. The cyclo­hexane ring adopts a chair conformation. In the crystal structure, N—H⋯O hydrogen bonds, augmented by weak C—H⋯O inter­actions, link the mol­ecules into transverse chains along *a*. These chains are linked into zigzag columns down *a* by C—H⋯F hydrogen bonds and C—H⋯π inter­actions.

## Related literature

For background see: Saeed *et al.* (2008 ▶). For related structures, see: Kobal *et al.* (1990 ▶); Chopra & Guru Row (2008 ▶); Donnelly *et al.* (2008 ▶); Hou *et al.* (2004 ▶); Saeed *et al.* (2008 ▶). For information on the Cambridge Structural Database, see: Allen (2002 ▶). For ring puckering parameters, see: Cremer & Pople (1975 ▶).
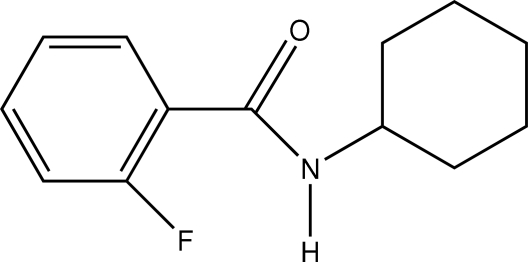


## Experimental

### 

#### Crystal data

C_13_H_16_FNO
*M* *_r_* = 221.27
Monoclinic, 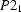

*a* = 5.1804 (6) Å
*b* = 6.5309 (8) Å
*c* = 16.6522 (19) Å
β = 91.336 (6)°
*V* = 563.24 (11) Å^3^
*Z* = 2
Mo *K*α radiation
μ = 0.09 mm^−1^
*T* = 91 (2) K
0.39 × 0.16 × 0.08 mm

#### Data collection

Bruker APEXII CCD area-detector diffractometer
Absorption correction: multi-scan (*SADABS*; Bruker, 2006 ▶) *T*_min_ = 0.822, *T*_max_ = 0.993
7905 measured reflections
2117 independent reflections
1884 reflections with *I* > 2σ(*I*)
*R*_int_ = 0.031

#### Refinement

*R*[*F* ^2^ > 2σ(*F* ^2^)] = 0.035
*wR*(*F* ^2^) = 0.114
*S* = 1.12
2117 reflections
148 parameters
1 restraint
H atoms treated by a mixture of independent and constrained refinement
Δρ_max_ = 0.30 e Å^−3^
Δρ_min_ = −0.26 e Å^−3^

### 

Data collection: *APEX2* (Bruker, 2006 ▶); cell refinement: *APEX2* and *SAINT* (Bruker, 2006 ▶); data reduction: *SAINT*; program(s) used to solve structure: *SHELXS97* (Sheldrick, 2008 ▶) and *TITAN* (Hunter & Simpson, 1999 ▶); program(s) used to refine structure: *SHELXL97* (Sheldrick, 2008 ▶) and *TITAN*; molecular graphics: *ORTEPIII* (Farrugia, 1997 ▶) and *Mercury* (Macrae *et al.*, 2006 ▶); software used to prepare material for publication: *SHELXL97*, *enCIFer* (Allen *et al.*, 2004 ▶), *PLATON* (Spek, 2003 ▶) and *publCIF* (Westrip, 2008 ▶).

## Supplementary Material

Crystal structure: contains datablocks global, I. DOI: 10.1107/S1600536808034090/lh2713sup1.cif

Structure factors: contains datablocks I. DOI: 10.1107/S1600536808034090/lh2713Isup2.hkl

Additional supplementary materials:  crystallographic information; 3D view; checkCIF report

## Figures and Tables

**Table 1:** Hydrogen-bond geometry (Å, °)

*D*—H⋯*A*	*D*—H	H⋯*A*	*D*⋯*A*	*D*—H⋯*A*
N1—H*N*1⋯O1^i^	0.87 (2)	2.20 (2)	3.0092 (18)	153.8 (19)
C9—H9*B*⋯O1^i^	0.99	2.67	3.446 (2)	136
C4—H4⋯F1^ii^	0.95	2.43	3.2326 (19)	142
C5—H5⋯*Cg*1^iii^	0.95	2.96	3.759 (2)	142
C9—H9*A*⋯*Cg*1^iv^	0.99	2.71	3.644 (2)	157

Symmetry codes: (i) 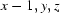; (ii) 
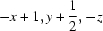
; (iii) 
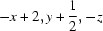
; (iv) 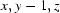. *Cg*1 is the centroid of the C2–C7 benzene ring.

**Fig. 1.**
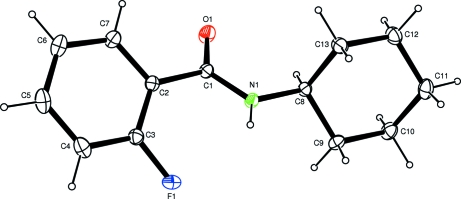
The structure of (I) with displacement ellipsoids for the non-hydrogen atoms drawn at the 50% probability level.

**Fig. 2.**
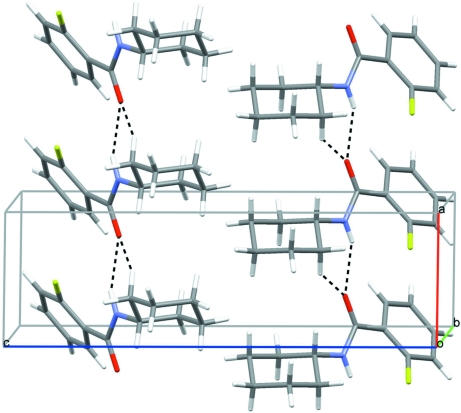
Transverse chains formed along a by N—H···O hydrogen bonds.

**Fig. 3.**
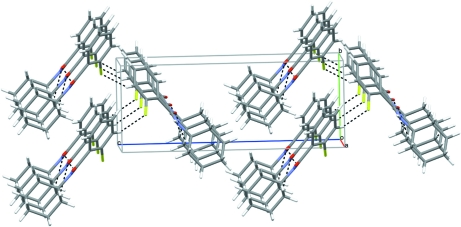
Crystal packing of (I) viewed down the a axis.

## supplementary crystallographic information

### Comment

The background to this study has been described in our earlier paper reporting the structure of 4-chloro-*N*-(3-methoxyphenyl)-benzamide (Saeed *et al.* 2008).

We report here the structure of the title 2-fluorobenzamide derivative, I, Fig 1. The C2—C1—O1—N1—C8 unit is planar with a maximum deviation of 0.0223 (10) Å. This plane makes a dihedral angle of 29.92 (7) ° with the fluorobenzene ring plane. The *N*-cyclohexyl ring adopts a chair conformation with Cremer-Pople puckering parameters Q(2)= 0.0138 (15) Å, φ(2) = 23 (2)° and Q(3) = 0.5763 (15)Å (Cremer & Pople, 1975). 2-fluorobenzamide derivatives with aliphatic substituents on the amide N atom are unusual with only one reasonably comparable derivative (Kobal *et al.* 1990) of the Cambridge Structural Database V5.29 (Allen, 2002). In contrast, *N*-aryl derivatives are more common and the salient bond distances and angles in the present molecule agree well with those reported previously (see for example Chopra & Guru Row, 2008; Donnelly *et al.*, 2008; Hou *et al.*, 2004).

In the crystal structure, chains are formed that run in opposite directions along *a* through N—H···O hydrogen bonds, Table 1, Fig 2. These interactions are supported by weak C9—H9B···O1 hydrogen bonds. Additional weak C—H···F hydrogen bonds and C—H···π interactions link these chains into zigzag columns down *a* Fig. 3.

### Experimental

2-Fluorobenzoyl chloride (1 mmol) in CHCl_3_ was treated with cyclohexyl amine (3.5 mmol) under a nitrogen atmosphere at reflux for 5 h. Upon cooling, the reaction mixture was diluted with CHCl_3_ and washed consecutively with 1 *M* aq HCl and saturated aq NaHCO_3_. The organic layer was dried over anhydrous sodium sulfate and concentrated under reduced pressure. Crystallization of the residue from CHCl_3_ afforded the title compound (79%) as white needles: Anal. calcd. for C_13_H_16_FNO: C 70.56, H 7.29, N 6.33%; found: C 70.08, H 7.31, N 6.38%.

### Refinement

The H atom bound to N1 was located in a difference electron density map and refined freely with an isotropic displacememt parameter. All other H-atoms were refined using a riding model with d(C—H) = 0.95 Å, *U*_iso_= 1.2*U*_eq_ (C) for aromatic and 0.98 Å, *U*_iso_ = 1.5*U*_eq_ (C) for CH_3_ H atoms. In the absence of significant anomalous dispersion effects, Friedel pairs were averaged.

### Crystal data

**Table:**

C_13_H_16_FNO	*F*(000) = 236
*M**_r_* = 221.27	*D*_x_ = 1.305 Mg m^−^^3^
Monoclinic, *P*2_1_	Mo *K*α radiation, λ = 0.71073 Å
Hall symbol: P 2yb	Cell parameters from 2700 reflections
*a* = 5.1804 (6) Å	θ = 3.4–32.5°
*b* = 6.5309 (8) Å	µ = 0.09 mm^−^^1^
*c* = 16.6522 (19) Å	*T* = 91 K
β = 91.336 (6)°	Needle, colourless
*V* = 563.24 (11) Å^3^	0.39 × 0.16 × 0.08 mm
*Z* = 2	

### Data collection

**Table:**

Bruker APEXII CCD area-detector diffractometer	2117 independent reflections
Radiation source: fine-focus sealed tube	1884 reflections with *I* > 2σ(*I*)
graphite	*R*_int_ = 0.031
ω scans	θ_max_ = 33.4°, θ_min_ = 1.2°
Absorption correction: multi-scan (SADABS; Bruker, 2006)	*h* = −7→7
*T*_min_ = 0.822, *T*_max_ = 0.993	*k* = −8→9
7905 measured reflections	*l* = −25→24

### Refinement

**Table:**

Refinement on *F*^2^	Primary atom site location: structure-invariant direct methods
Least-squares matrix: full	Secondary atom site location: difference Fourier map
*R*[*F*^2^ > 2σ(*F*^2^)] = 0.036	Hydrogen site location: inferred from neighbouring sites
*wR*(*F*^2^) = 0.114	H atoms treated by a mixture of independent and constrained refinement
*S* = 1.12	*w* = 1/[σ^2^(*F*_o_^2^) + (0.0702*P*)^2^ + 0.0113*P*] where *P* = (*F*_o_^2^ + 2*F*_c_^2^)/3
2117 reflections	(Δ/σ)_max_ = 0.002
148 parameters	Δρ_max_ = 0.30 e Å^−^^3^
1 restraint	Δρ_min_ = −0.26 e Å^−^^3^

### Special details

**Table:**

Geometry. All e.s.d.'s (except the e.s.d. in the dihedral angle between two l.s. planes) are estimated using the full covariance matrix. The cell e.s.d.'s are taken into account individually in the estimation of e.s.d.'s in distances, angles and torsion angles; correlations between e.s.d.'s in cell parameters are only used when they are defined by crystal symmetry. An approximate (isotropic) treatment of cell e.s.d.'s is used for estimating e.s.d.'s involving l.s. planes.
Refinement. Refinement of *F*^2^ against ALL reflections. The weighted *R*-factor *wR* and goodness of fit *S* are based on *F*^2^, conventional *R*-factors *R* are based on *F*, with *F* set to zero for negative *F*^2^. The threshold expression of *F*^2^ > σ(*F*^2^) is used only for calculating *R*-factors(gt) *etc*. and is not relevant to the choice of reflections for refinement. *R*-factors based on *F*^2^ are statistically about twice as large as those based on *F*, and *R*- factors based on ALL data will be even larger.

### Fractional atomic coordinates and isotropic or equivalent isotropic displacement parameters (Å^2^)

**Table:**

	*x*	*y*	*z*	*U*_iso_*/*U*_eq_	
O1	1.2369 (2)	0.9202 (2)	0.24742 (7)	0.0187 (3)	
C1	1.0126 (3)	0.9513 (2)	0.22274 (9)	0.0122 (3)	
C2	0.9598 (3)	1.1225 (2)	0.16419 (9)	0.0124 (3)	
C3	0.7571 (3)	1.1292 (2)	0.10763 (9)	0.0142 (3)	
F1	0.5940 (2)	0.96735 (18)	0.10014 (5)	0.0207 (2)	
C4	0.7162 (3)	1.2929 (3)	0.05603 (9)	0.0187 (3)	
H4	0.5765	1.2913	0.0180	0.022*	
C5	0.8825 (3)	1.4593 (3)	0.06068 (10)	0.0207 (3)	
H5	0.8553	1.5740	0.0264	0.025*	
C6	1.0902 (3)	1.4580 (3)	0.11584 (10)	0.0203 (3)	
H6	1.2055	1.5711	0.1188	0.024*	
C7	1.1270 (3)	1.2908 (3)	0.16621 (9)	0.0162 (3)	
H7	1.2697	1.2906	0.2031	0.019*	
N1	0.8095 (3)	0.8430 (2)	0.24730 (8)	0.0129 (3)	
HN1	0.655 (4)	0.883 (4)	0.2321 (12)	0.015*	
C8	0.8378 (3)	0.6807 (2)	0.30746 (9)	0.0120 (3)	
H8	1.0140	0.6199	0.3028	0.014*	
C9	0.6393 (3)	0.5126 (2)	0.29046 (10)	0.0144 (3)	
H9A	0.6632	0.4574	0.2358	0.017*	
H9B	0.4631	0.5708	0.2927	0.017*	
C10	0.6683 (3)	0.3397 (3)	0.35200 (10)	0.0181 (3)	
H10A	0.8385	0.2730	0.3462	0.022*	
H10B	0.5329	0.2354	0.3416	0.022*	
C11	0.6456 (3)	0.4213 (3)	0.43778 (10)	0.0193 (3)	
H11A	0.4685	0.4736	0.4455	0.023*	
H11B	0.6765	0.3084	0.4765	0.023*	
C12	0.8402 (3)	0.5932 (3)	0.45444 (10)	0.0184 (3)	
H12A	0.8131	0.6494	0.5088	0.022*	
H12B	1.0175	0.5368	0.4532	0.022*	
C13	0.8128 (3)	0.7651 (3)	0.39258 (9)	0.0167 (3)	
H13A	0.9481	0.8695	0.4029	0.020*	
H13B	0.6425	0.8319	0.3977	0.020*	

### Atomic displacement parameters (Å^2^)

**Table:**

	*U*^11^	*U*^22^	*U*^33^	*U*^12^	*U*^13^	*U*^23^
O1	0.0108 (5)	0.0210 (6)	0.0242 (6)	−0.0001 (4)	−0.0012 (4)	0.0060 (5)
C1	0.0126 (6)	0.0105 (6)	0.0135 (6)	0.0003 (5)	0.0004 (5)	0.0002 (5)
C2	0.0123 (6)	0.0121 (6)	0.0127 (6)	0.0005 (5)	0.0017 (5)	0.0002 (5)
C3	0.0137 (6)	0.0154 (7)	0.0134 (6)	−0.0003 (6)	0.0008 (5)	0.0008 (6)
F1	0.0216 (5)	0.0223 (5)	0.0178 (4)	−0.0069 (4)	−0.0065 (4)	0.0005 (4)
C4	0.0184 (7)	0.0235 (8)	0.0143 (6)	0.0043 (7)	0.0022 (5)	0.0036 (6)
C5	0.0218 (7)	0.0200 (8)	0.0207 (7)	0.0060 (6)	0.0061 (6)	0.0080 (7)
C6	0.0204 (7)	0.0151 (7)	0.0256 (8)	−0.0007 (6)	0.0061 (6)	0.0043 (7)
C7	0.0151 (7)	0.0149 (7)	0.0186 (7)	−0.0016 (6)	0.0012 (5)	0.0012 (6)
N1	0.0103 (5)	0.0135 (6)	0.0149 (6)	−0.0007 (5)	−0.0013 (4)	0.0032 (5)
C8	0.0114 (6)	0.0108 (6)	0.0136 (6)	0.0003 (5)	−0.0010 (5)	0.0021 (5)
C9	0.0133 (6)	0.0115 (7)	0.0183 (7)	−0.0011 (5)	−0.0006 (5)	−0.0004 (5)
C10	0.0191 (7)	0.0121 (7)	0.0230 (8)	−0.0011 (6)	−0.0002 (6)	0.0026 (6)
C11	0.0188 (7)	0.0177 (8)	0.0215 (7)	0.0016 (6)	0.0029 (6)	0.0071 (6)
C12	0.0224 (7)	0.0175 (8)	0.0152 (7)	0.0007 (6)	−0.0011 (6)	0.0028 (6)
C13	0.0234 (7)	0.0122 (7)	0.0142 (6)	−0.0008 (6)	−0.0023 (6)	0.0004 (5)

### Geometric parameters (Å, °)

**Table:**

O1—C1	1.2402 (18)	C8—C13	1.529 (2)
C1—N1	1.3395 (19)	C8—H8	1.0000
C1—C2	1.504 (2)	C9—C10	1.530 (2)
C2—C3	1.395 (2)	C9—H9A	0.9900
C2—C7	1.399 (2)	C9—H9B	0.9900
C3—F1	1.3571 (19)	C10—C11	1.532 (2)
C3—C4	1.384 (2)	C10—H10A	0.9900
C4—C5	1.388 (3)	C10—H10B	0.9900
C4—H4	0.9500	C11—C12	1.530 (2)
C5—C6	1.398 (2)	C11—H11A	0.9900
C5—H5	0.9500	C11—H11B	0.9900
C6—C7	1.387 (2)	C12—C13	1.528 (2)
C6—H6	0.9500	C12—H12A	0.9900
C7—H7	0.9500	C12—H12B	0.9900
N1—C8	1.4632 (19)	C13—H13A	0.9900
N1—HN1	0.87 (2)	C13—H13B	0.9900
C8—C9	1.526 (2)		
			
O1—C1—N1	123.26 (14)	C8—C9—C10	110.57 (12)
O1—C1—C2	119.42 (13)	C8—C9—H9A	109.5
N1—C1—C2	117.29 (12)	C10—C9—H9A	109.5
C3—C2—C7	116.58 (14)	C8—C9—H9B	109.5
C3—C2—C1	125.69 (14)	C10—C9—H9B	109.5
C7—C2—C1	117.74 (13)	H9A—C9—H9B	108.1
F1—C3—C4	117.29 (13)	C9—C10—C11	111.03 (14)
F1—C3—C2	119.65 (14)	C9—C10—H10A	109.4
C4—C3—C2	123.03 (15)	C11—C10—H10A	109.4
C3—C4—C5	118.96 (14)	C9—C10—H10B	109.4
C3—C4—H4	120.5	C11—C10—H10B	109.4
C5—C4—H4	120.5	H10A—C10—H10B	108.0
C4—C5—C6	119.94 (15)	C12—C11—C10	111.09 (14)
C4—C5—H5	120.0	C12—C11—H11A	109.4
C6—C5—H5	120.0	C10—C11—H11A	109.4
C7—C6—C5	119.64 (16)	C12—C11—H11B	109.4
C7—C6—H6	120.2	C10—C11—H11B	109.4
C5—C6—H6	120.2	H11A—C11—H11B	108.0
C6—C7—C2	121.84 (14)	C13—C12—C11	111.48 (13)
C6—C7—H7	119.1	C13—C12—H12A	109.3
C2—C7—H7	119.1	C11—C12—H12A	109.3
C1—N1—C8	121.65 (12)	C13—C12—H12B	109.3
C1—N1—HN1	118.4 (16)	C11—C12—H12B	109.3
C8—N1—HN1	119.3 (15)	H12A—C12—H12B	108.0
N1—C8—C9	109.73 (12)	C12—C13—C8	110.57 (13)
N1—C8—C13	111.36 (12)	C12—C13—H13A	109.5
C9—C8—C13	111.09 (13)	C8—C13—H13A	109.5
N1—C8—H8	108.2	C12—C13—H13B	109.5
C9—C8—H8	108.2	C8—C13—H13B	109.5
C13—C8—H8	108.2	H13A—C13—H13B	108.1
			
O1—C1—C2—C3	152.45 (16)	C1—C2—C7—C6	−178.45 (14)
N1—C1—C2—C3	−29.4 (2)	O1—C1—N1—C8	1.5 (2)
O1—C1—C2—C7	−27.8 (2)	C2—C1—N1—C8	−176.60 (13)
N1—C1—C2—C7	150.43 (14)	C1—N1—C8—C9	−147.62 (14)
C7—C2—C3—F1	177.27 (13)	C1—N1—C8—C13	88.97 (17)
C1—C2—C3—F1	−2.9 (2)	N1—C8—C9—C10	179.13 (12)
C7—C2—C3—C4	−0.8 (2)	C13—C8—C9—C10	−57.29 (16)
C1—C2—C3—C4	179.00 (14)	C8—C9—C10—C11	56.43 (17)
F1—C3—C4—C5	−178.59 (14)	C9—C10—C11—C12	−55.36 (18)
C2—C3—C4—C5	−0.5 (2)	C10—C11—C12—C13	55.12 (19)
C3—C4—C5—C6	1.2 (2)	C11—C12—C13—C8	−55.66 (18)
C4—C5—C6—C7	−0.7 (3)	N1—C8—C13—C12	179.44 (13)
C5—C6—C7—C2	−0.7 (2)	C9—C8—C13—C12	56.80 (17)
C3—C2—C7—C6	1.4 (2)		

### Hydrogen-bond geometry (Å, °)

**Table:**

*D*—H···*A*	*D*—H	H···*A*	*D*···*A*	*D*—H···*A*
N1—HN1···O1^i^	0.87 (2)	2.20 (2)	3.0092 (18)	153.8 (19)
C9—H9B···O1^i^	0.99	2.67	3.446 (2)	136
C4—H4···F1^ii^	0.95	2.43	3.2326 (19)	142
C5—H5···Cg1^iii^	0.95	2.96	3.759 (2)	142
C9—H9A···Cg1^iv^	0.99	2.71	3.644 (2)	157

Symmetry codes: (i) *x*−1, *y*, *z*; (ii) −*x*+1, *y*+1/2, −*z*; (iii) −*x*+2, *y*+1/2, −*z*; (iv) *x*, *y*−1, *z*.
